# Low oxygen saturation during sleep reduces CD1D and RAB20 expressions that are reversed by CPAP therapy

**DOI:** 10.1016/j.ebiom.2020.102803

**Published:** 2020-06-05

**Authors:** Tamar Sofer, Ruitong Li, Roby Joehanes, Honghuang Lin, Adam C. Gower, Heming Wang, Nuzulul Kurniansyah, Brian E. Cade, Jiwon Lee, Stephanie Williams, Reena Mehra, Sanjay R. Patel, Stuart F. Quan, Yongmei Liu, Jerome I. Rotter, Stephen S. Rich, Avrum Spira, Daniel Levy, Sina A. Gharib, Susan Redline, Daniel J. Gottlieb

**Affiliations:** a Division of Sleep Medicine, Harvard Medical School, Boston, MA 02115, USA; b Division of Sleep and Circadian Disorders, Department of Medicine, Brigham and Women's Hospital, Boston, MA, USA; c Department of Biostatistics, Harvard T. H. Chan School of Public Health, Boston, MA, USA; d The Population Sciences Branch of the National Heart, Lung and Blood Institute, Bethesda, MD, USA and the Framingham Heart Study, Framingham, MA, USA; e Hebrew SeniorLife, Harvard Medical School, Boston, MA, USA; f Department of Medicine, Boston University School of Medicine, Boston, MA, USA; g Clinical and Translational Science Institute, Boston University School of Medicine, Boston, USA; h Neurologic Institute, Cleveland Clinic, Cleveland, OH, USA; i Division of Pulmonary, Allergy and Critical Care Medicine, University of Pittsburgh, Pittsburgh, PA, USA; j Department of Medicine, Duke University School of Medicine, Durham, NC, USA; k The Institute for Translational Genomics and Population Sciences, Departments of Pediatrics and Medicine, Los Angeles Biomedical Research Institute at Harbor-UCLA Medical Center, Torrance, CA, USA; l Center for Public Health Genomics, Department of Public Health Sciences, University of Virginia, Charlottesville, VA, USA; m Boston University School of Medicine and Boston Medical Center, Boston, MA, USA; n Computational Medicine Core, Center for Lung Biology, University of Washington Medicine Sleep Center, Division of Pulmonary, Critical Care, and Sleep Medicine, University of Washington, Seattle, WA, USA; o Division of Pulmonary, Critical Care, and Sleep Medicine, Beth Israel Deaconess Medical Center, Boston, MA, USA; p VA Boston Healthcare System, Boston, MA, USA

**Keywords:** Gene expression, Obstructive Sleep Apnea, Hypoxemia

## Abstract

**Background:**

Sleep Disordered Breathing (SDB) is associated with a wide range of pathophysiological changes due, in part, to hypoxemia during sleep. We sought to identify gene transcription associations with measures of SDB and hypoxemia during sleep, and study their response to treatment.

**Methods:**

In two discovery cohorts, Framingham Offspring Study (FOS; *N* = 571) and the Multi-Ethnic Study of Atherosclerosis (MESA; *N* = 580), we studied gene expression in peripheral blood mononuclear cells in association with three measures of SDB: Apnea Hypopnea Index (AHI); average oxyhemoglobin saturation (avgO2) during sleep; and minimum oxyhemoglobin saturation (minO2) during sleep. Associated genes were used for analysis of gene expression in the blood of 15 participants with moderate or severe obstructive sleep apnea (OSA) from the Heart Biomarkers In Apnea Treatment (HeartBEAT) trial. These genes were studied pre- and post-treatment (three months) with continuous positive airway pressure (CPAP). We also performed Gene Set Enrichment Analysis (GSEA) on all traits and cohort analyses.

**Findings:**

Twenty-two genes were associated with SDB traits in both MESA and FOS. Of these, lower expression of *CD1D* and *RAB20* was associated with lower avgO2 in MESA and FOS. CPAP treatment increased the expression of these genes in HeartBEAT participants. Immunity and inflammation pathways were up-regulated in subjects with lower avgO2; *i.e*., in those with a more severe SDB phenotype (MESA), whereas immuno-inflammatory processes were down-regulated following CPAP treatment (HeartBEAT).

**Interpretation:**

Low oxygen saturation during sleep is associated with alterations in gene expression and transcriptional programs that are partially reversed by CPAP treatment.

**Research in Context**

**Evidence before this study**

Obstructive sleep apnea (OSA) is a common but complex and heterogeneous disorder, characterized by intermittent breathing pauses during sleep that can result in reduced oxygen saturation in blood. Repeated cycles of desaturation and re-oxygenation can activate circulating white blood cells and are associated with endothelial dysfunction. However, the pathogenetic mechanisms elicited by OSA remain largely unknown. The most effective treatment for OSA is continuous positive airway pressure (CPAP). Currently, only small studies with limited number of individuals have investigated the transcriptional consequences of OSA on peripheral blood leukocytes and the response to CPAP therapy.

**Added value of this study**

We conducted the largest study to date, utilizing sleep studies in over one thousand subjects, to link peripheral blood transcriptional signals with OSA traits, including oxygen saturation during sleep. We found multiple molecular mechanisms activated in OSA that were primarily related to immune and inflammatory pathways, and identified candidate genes associated with hypoxemia during sleep. By investigating changes in gene expression in the blood of fifteen individuals with OSA before and after three months of CPAP therapy, we demonstrated that some of these processes are reversed by treatment.

**Implications of all the available evidence**

Leveraging the compendium of genes and pathways identified in our work can: i) lead to further investigation into molecular mechanisms involved in the pathogenesis of OSA; ii) guide rational targeting of specific pathways and candidate genes to alter the adverse course of untreated OSA; and iii) help develop potential biomarkers of OSA for which CPAP is an effective treatment.

Alt-text: Unlabelled box

## 1 Introduction

Sleep Disordered Breathing (SDB) is characterized by abnormal respiratory patterns during sleep and is usually associated with impaired gas exchange. The most common type, Obstructive Sleep Apnea (OSA), is estimated to be prevalent in 17% of women and 34% of men, aged 30–70 years [1], and contributes significantly to the incidence and morbidity of cardio-metabolic disease, including hypertension, stroke, heart failure, coronary artery disease, atrial fibrillation, and diabetes [2], [3], [4], [5], [6], [7], [8]. OSA is characterized by episodic complete (apneas) or partial (hypopneas) cessation of breathing during sleep, resulting in intermittent hypercapnic hypoxemia. The molecular mechanisms underlying OSA-mediated physiological stress are not well established, but include genetic and gene regulatory factors.

Hypoxemia is known to influence gene expression, partly by affecting transcription factor activation, including both HIF-1α and NF-κB [9]. Intermittent hypoxemia has been shown to affect gene expression in peripheral blood mononuclear cells of healthy volunteers [10]. Six studies ranging in size from 18 to 48 individuals have evaluated the impact of OSA on gene expression in peripheral blood monocytes or total leukocytes, either cross-sectionally in comparison to those without OSA [10], [11], [12], or pre- and post-treatment with continuous positive airway pressure (CPAP; [13,14]), the most common therapy for OSA, or following two weeks of CPAP withdrawal into either sham or oxygen supplement treatment [15]. In addition to their small sample sizes, some of these studies were limited by targeting a specific patient population (*e.g*., children; [12]), a limited set of genes [11], and by a focus on dichotomized (presence/absence) OSA traits.

We conducted the largest analysis to date of genome-wide transcriptional associations with multiple traits that characterize SDB-related hypoxemia: the apnea hypopnea index (AHI), the average oxyhemoglobin saturation during sleep (avgO2), and the minimum oxyhemoglobin saturation during sleep (minO2), respectively capturing intermittent hypoxemia and mean and maximal severity of hypoxemia. We first performed a discovery association analysis of gene expression in two population-based cohorts: the Framingham Offspring Study (FOS; [16]) and the Multi-Ethnic Study of Atherosclerosis (MESA; [17]). We further evaluated genes identified in the discovery study using fifteen individuals from the Heart Biomarkers in Apnea Treatment (HeartBEAT) clinical trial [18]. These subjects had moderate to severe OSA and were adherent to CPAP for three months. We then performed Gene Set Enrichment Analysis (GSEA) in each of these cohorts to determine those processes that are associated with distinct SDB traits and, in the case of HeartBEAT, we assessed the effect of OSA therapy in altering canonical biological pathways.

## 2 Materials and methods

**Fig. 1:**
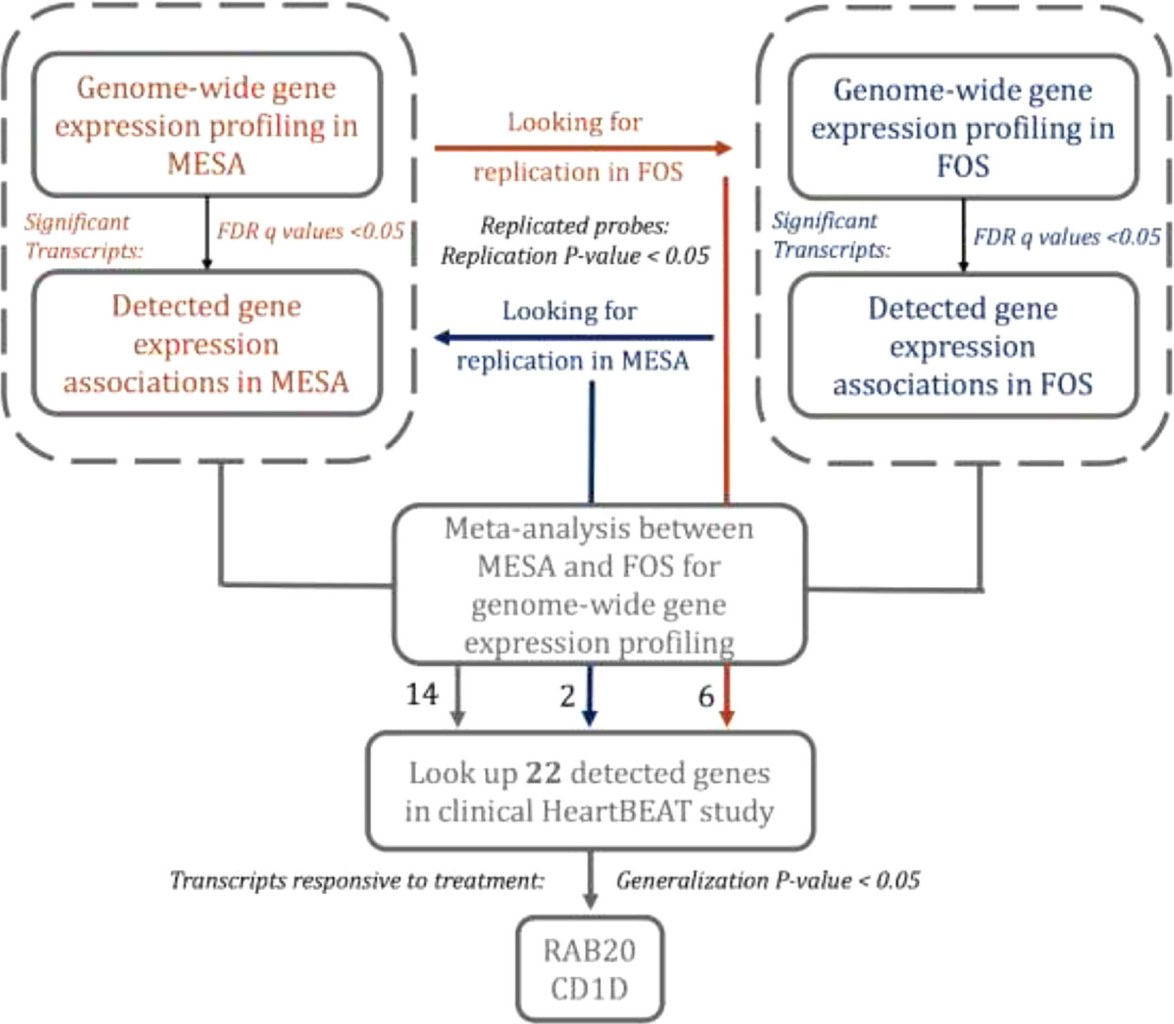
**Gene-based analysis workflow**. This figure displays the cross-replication and meta-analysis approach for discovery of gene transcripts associated with OSA traits, and follow-up association analysis of response to CPAP treatment in HeartBEAT.

The central study design is illustrated in Fig. 1. We started with a single-gene association discovery step, in which we studied the association of gene-expression (outcomes) with SDB traits (exposures) in two discovery cohorts: the Framingham Offspring Study (FOS) and the Multi-Ethnic Study of Atherosclerosis (MESA). First, we performed association analysis in each of FOS and MESA separately, and tested for replication of gene expression associations in the second study (cross-replication), following the standard in genetic analysis for performing replication for limiting false discoveries. Then, to increase power, we meta-analyzed estimated associations across FOS and MESA. Finally, we took genes that either cross-replicated between FOS and MESA or were detected in their meta-analysis and studied whether the expression of these genes responded to CPAP treatment in the HeartBEAT clinical study. MESA, FOS, and HeartBEAT study were approved by Institutional Review Boards in all study centers and participants provided written informed consent. Below, we first define the sleep parameters measured, then describe the FOS, MESA, and HeartBEAT studies. For each cohort, we outline the statistical methods used for evaluating gene expression profiles and their association with SDB traits.

### 2.1 SDB phenotypes and covariates

In both discovery cohorts, we studied three quantitative measures of SDB, assessed by overnight polysomnography (PSG): 1) Apnea-Hypopnea index (AHI), defined as the number of apneas plus hypopneas per hour of sleep that were associated with at least 3% oxygen desaturation from the preceding baseline; 2) Average oxyhemoglobin saturation during sleep (avgO2); and 3) Minimum oxyhemoglobin saturation during sleep (minO2). These complementary phenotypes capture three distinct aspects of oxygen saturation. AHI is the most common metric used to characterize OSA and reflects intermittent hypoxemia, a known trigger of sympathetic activation and oxidative stress. Average oxygen saturation is one of the most heritable OSA traits [19], and there is evidence that its impact on gene expression may differ from that of intermittent hypoxemia [20]. Minimum oxyhemoglobin saturation reflects the maximal severity of hypoxemia, and may allow identification of genes responsive to extremes of oxyhemoglobin desaturation. Covariates included sex, age, and BMI (for sensitivity analysis).

### 2.2 The multi-ethnic study of atherosclerosis (MESA)

MESA is a longitudinal cohort study, established in 2000, that prospectively collected risk factors for development of cardiovascular disease among participants in six field centers across the United States (Baltimore City and Baltimore County, MD; Chicago, IL; Forsyth County, NC; Los Angeles County, CA; Northern Manhattan and the Bronx, NY; and St. Paul, MN). The cohort has been studied every few years. The present analysis considers *N* = 619 individuals who participated in a sleep study [17,21] and had available gene expression data. Blood for gene expression was drawn during the Exam 5 core examination (2010–2012) and sleep studies were conducted in an ancillary exam (2010–2013). Sleep data were collected using standardized full in-home level-2 polysomnography (Compumedics Somte Systems, Abbotsville, Australia, AU0), as described before [21]. After removal of 39 observations due to missing sleep data, we included 580 participants in the analysis: 196 African-Americans (AA), 259 European-Americans (EA) and 125 Hispanic-Europeans (HA). Gene expression data collection and processing in MESA are described in the Supplementary Materials.

### 2.3 Association analysis in MESA

For each gene, we fit linear mixed models separately in each race/ethnic group to estimate the association between SDB exposures and gene expression markers, with a random effect for gene expression chip. Covariates (fixed effects) were study site, residual cell contamination (Neutrophils, Natural Killer cells, T cell, B cell), age, and sex. When the model did not converge due to null variance component, we fit a linear regression model with original fixed effects. The regression results from the three groups were then combined using inverse-variance fixed effects meta-analysis. In sensitivity analysis, we also adjusted for BMI.

### 2.4 Framingham offspring study (FOS)

FOS, initiated in 1971, is a prospective epidemiologic study of 5124 young adults, consisting of offspring of the original Framingham Heart Study cohort and their recruited spouses [22]. The cohort has been examined every 4–8 years for standardized medical history, blood tests and 12-lead ECG [16,23]. Of FOS participants, 699 were enrolled in the Sleep Heart Health Study (SHHS), which was initiated with a first visit (1995 – 1998) that included in-home level-2 polysomnography (Compumedics P-Series, Abbotsville, Australia), followed by a second visit (2001 – 2003), in which a subset of 385 people underwent repeat polysomnography [24]. At the FOS eighth examination cycle (2005 - 2008), RNA was extracted from whole blood for gene expression profiling [25]. Here, we consider 517 European-Americans individuals with both gene expression and polysomnography data. To minimize the time gap between gene expression and polysomnography measurements and maximize statistical power, the polysomnography measurements and corresponding phenotypes were taken from the second SHHS visit when available, and otherwise, from the first SHHS visit. Gene expression data collection and processing in FOS are described in the Supplementary Materials.

### 2.5 Association analysis in FOS

For each gene, we fit a linear mixed model to estimate the association between SDB exposure and gene expression markers, with a random effect accounting for familial relatedness. Covariates (fixed effects) were estimated white blood cell counts (Neutrophils, Natural Killer cells, T cell, B cell), sex, age, and in sensitivity analysis, BMI. Because for FOS the expression measures were taken from blood 2–13 years after the sleep study, it is not clear how and if time varying covariates may confound the SDB-trait gene expression association, and we adjusted for age and BMI measures at the time of the sleep study.

### 2.6 Cross-replication analysis of FOS and MESA

Each of the MESA and FOS studies where treated as both a “discovery” and a “replication” study. For each discovery cohort and each of the SDB exposures, we computed FDR-adjusted *p* values (FDR *q* values; [26]) across the expression transcripts. Then, we took all expression transcripts with *q* < 0.05 for replication in the other cohort. Replication is complicated by the fact that there are sometimes multiple transcripts per gene, and further, MESA and FOS used different platforms, so that gene transcripts did not necessarily match between the discovery and replication studies. Therefore, we used an approach termed “many to many” [27] in which a transcript of a specific gene in the discovery study was matched to all transcripts of the same gene in the replication study. After matching the list of discovery transcripts with FDR *q* < 0.05 in one cohort to a list of transcripts in the second cohort, a corresponding gene was replicated if one of the transcripts in the replication study had a one-sided *p* value < 0.05, requiring that the direction of association be the same as the transcript in the discovery study [28]. Replicated genes were carried forward for further analysis in the CPAP treatment cohort.

### 2.7 Meta-analysis of FOS and MESA

Using the same “many-to-many” approach described above, we matched all expression transcripts in MESA and FOS, and performed inverse-variance fixed-effects meta-analysis for overall exposure effect. We computed FDR-controlling *q*s. Genes with at least one transcript with FDR-value < 0.05 were carried forward for further analysis in the CPAP treatment cohort.

### 2.8 Treatment response in a clinical cohort: the heart biomarker evaluation in apnea treatment (HeartBEAT) study

The Heart Biomarker Evaluation in Apnea Treatment (HeartBEAT) study is a randomized, 4-site single-blind clinical trial that investigated the efficacy of OSA therapy in reducing cardiovascular disease risk for patients with moderate-severe OSA (ClinicalTrials.gov NCT01086800; [18]). Of HeartBEAT participants randomized to the CPAP treatment group, a subsample of 15 individuals who were highly adherent to CPAP therapy (defined by at least four hours of CPAP use per night over the three-month intervention period) participated in gene expression analysis. Blood was drawn in the morning after fasting, prior to initiation of CPAP and at the end of the 12-week treatment period.

### 2.9 Gene expression analysis in heartbeat

Venipuncture was performed in the morning following an overnight fast. Venous blood was collected in 8 mL heparinized Cell Prep Tubes containing Ficoll Hypaque (Becton Dickinson #362,753) in order to separate peripheral blood mononuclear cells. The tubes were centrifuged fresh at room temperature for 15 min at 2000 G to isolate the buffy coat, which was pelleted, resuspended in Millipore S-002–10F freezing medium, and cryopreserved at −80 C. Total RNA was extracted using Qiagen's miRNeasy Mini kit following the manufacturer's standard protocol. Gene expression profiling was performed using the Affymetrix Human Gene 1.0 ST array in samples collected before initiating CPAP and after three months of CPAP treatment. Raw CEL files were normalized to produce gene-level expression values using the implementation of the Robust Multiarray Average (RMA) in the affy Bioconductor R package (version 1.36.1) and an Entrez Gene-specific probeset mapping from the Molecular and Behavioral Neuroscience Institute (Brainarray) at the University of Michigan (version 16.0.0). For each gene, Student's paired *t*-test using expression values pre- and post-treatment in each individual was performed using the implementation in the multtest R package (version 2.14.0). Analysis of the HeartBEAT microarray data was performed using the R environment for statistical computing (version 2.15.1).

### 2.10 Testing of genes identified by MESA and FOS analyses

We used a statistical replication testing framework to study whether genes associated with SDB traits respond to OSA treatment using CPAP. We calculated the mean fold change with gene expression data collected post- and pre-CPAP therapy across the 15 HeartBEAT subjects. For any given gene that was carried forward from either the cross-replication or the meta-analysis of MESA and FOS (see Fig. 1), we computed the replication *p*-value by applying a one-sided *t*-test on the log of the estimated fold expression change. Because the replication framework requires that effects are consistent in their direction in the discovery and the replication stage, we defined the direction of association between gene expression marker and OSA as positive if the mean fold change was larger than 1 and negative otherwise. Thus, we computed one-sided *p*-values so that a gene could be declared as replicated in HeartBEAT only if its expression was positively associated with more severe SDB in MESA and FOS and decreased following three months of CPAP treatment in HeartBEAT (or vice versa). Finally, a gene was determined as responding to treatment if its replication (one-sided) *p*-value was < 0.05.

### 2.11 Gene set enrichment analysis

We performed gene set enrichment analysis (GSEA, [29]) for quantitative SDB traits in each cohort. For MESA and FOS, GSEA was applied separately for AHI, avgO2, and minO2 using the entire gene expression profiles rank ordered based on each gene's test statistic for the corresponding SDB trait. For HeartBEAT, the transcriptome was rank ordered based on the paired *t* statistic computed with respect to CPAP therapy. When more than one transcript was available for a single gene (for MESA and FOS), a single transcript was selected at random. To maximize biological relevance, we leveraged only curated pathways (Molecular Signature Database “Canonical pathways” and “Hallmark” collections, gene symbol identifiers, version 6.0) for a total of 1379 gene sets, and performed permutation analysis (*n* = 1000 permutations). We used cutoff of FDR *q* < 0.05 to identify significantly enriched pathways. We used Enrichment Map [30], a plug-in application within the Cytoscape (v3.3.1) environment [31], to develop a network-based visualization of the GSEA results. Gene set pairs were connected to each other based on the overlap among their gene members (25% or greater).

### 2.12 Data statement

All summary statistics from analysis reported in this manuscript are provided in the Supplementary Material. Raw gene expression data for FOS are a part of SABRe CVD study on dbGaP, with dbGaP study accession: phs000363.v17.p11. Data can be obtained by requesting authorized access to the Framingham Cohort Study phs000007 on dbGaP. Gene expression data for MESA can be obtained using application to the MESA https://www.mesa-nhlbi.org. Raw and processed gene expression data for HeartBEAT have been deposited in the Gene Expression Omnibus (GEO), Series GSE133601.

## 3 Results

### 3.1 Participant characteristics

Demographic characteristics of the discovery studies FOS and MESA and the clinical intervention study HeartBEAT are provided in Table 1. Age and BMI were similar across cohorts. HeartBEAT study participants, who were recruited based on a minimum AHI of 15 events/hour, were somewhat heavier and had more severe OSA than the general population samples of FOS and MESA, and were also almost entirely male. FOS and HeartBEAT participants were almost all of European descent, while the MESA participants included African and Hispanic/Latino ancestry. Fig. 1 summarizes the analysis workflow.

**Table 1:** Characteristics of participants in the MESA, FOS, and HeartBEAT studies. For continuous variables, the table provides mean and standard deviation (in parentheses). For categorical variables, the table provides number of participants and percent of cohort (in parentheses). FOS characteristics are at the time of the sleep study. HeartBEAT characteristics are prior to CPAP treatment, except for CPAP adherence, which provides the average number of hours used by the participant per night over the three-month treatment period. Obesity is defined as BMI ≥ 30.

Characteristics	Cohort		
	MESA	FOS	HeartBEAT
N	619	517	15
**Demographics**			
Age (yr)	68·7 (9·2)	62·2 (9·0)	65·9 (7·7)
Male sex (%)	329 (53·2)	264 (51·1)	14 (93·3)
Ethnicity (%)			–
African Americans (%)	130 (21·0)	–	–
Hispanic Americans (%)	203 (32·8)	–	–
European Americans (%)	286 (46·2)	517 (100·0)	13 (86·7)
Mixed (%)	–	–	2 (13·3)
BMI (kg/m^2^)	29·8 (5·6)	29·0 (5·3)	31·5 (3·3)
Obesity (%)	270 (43·3)	175 (33·8)	8 (53·3)
**OSA Traits**			
avgO2 (%)	94·2 (1·8)	94·3 (2·0)	93·3 (1·7)
minO2 (%)	83·0 (7·7)	84·5 (6·2)	77·7 (7·2)
AHI (events/ hr)	19·6 (18·9)	14·4 (15·1)	36·9 (9·4)
CPAP adherence (hr/night)	–	–	5·7 (1·9)

### 3.2 Gene-based identification of transcripts associated with SDB traits

To identify differentially expressed genes associated with SDB traits, we first performed a cross-replication analysis, in which we conducted discovery analysis in each of FOS and MESA, followed by replication analysis in the other cohort. We then meta-analyzed the information from FOS and MESA to increase power [32].

Table 2 lists eight genes that associated with the SDB traits in the cross-replication analysis. The expression of *RAB20* was positively associated with avgO2 (i.e., negatively associated with OSA severity), while expression of *CDYL* was negatively associated with avgO2, in both FOS and MESA. Moreover, *RAB20* expression increased following CPAP treatment in HeartBEAT (*p* = 0·046). The expression of *TUBB6, INVS1ABP, MAPK1, VIM, STX2*, and *CRIP1* was associated with either minO2 or avgO2 in both MESA and FOS, but their expression was unchanged following CPAP treatment in HeartBEAT.

**Table 2:** Genes identified in cross-replication analysis of MESA and FOS. Each row represents a gene transcript that replicated between FOS and MESA. Discovery cohort is the study in which the expression transcript was discovered (FDR *q <* 0·05). The identifiers corresponding to the transcript within each gene expression platform (MESA probe, FOS probeset, HeartBEAT Entrez Gene ID) are indicated. Discovery *p* is the original discovery *p* value, prior to multiple-testing adjustment. Discovery FDR-value is the FDR *q* in the discovery study, after FDR correction was applied to the discovery trait analysis. Replication *p* values are one-sided *p* values in the follow-up cohorts, with directions based on the direction of association in the discovery cohort. Cross-replication refers to FOS, when MESA is the discovery study, and to MESA, when FOS is the discovery study. Sign is the direction of estimated association, with a positive (negative) sign representing higher (lower) expression with increasing trait value. Complete summary statistics from association analysis are provided in Supplementary files E3 (MESA) E4 (FOS), and E6 (HeartBEAT).

MESA Probe	FOS Probeset	HeartBEAT Entrez Gene ID	Gene	Discovery					Replication *p*	
				Cohort	Trait	Sign	*p*	FDR *q*	Cross-Replication	Heart BEAT
ILMN_1708881	3525498	*55647*	*RAB20*	FOS	avgO2	+	1·22E-07	2·17E-03	0·037	0·046
ILMN_1678075	2892979	*9425*	*CDYL*	FOS	avgO2	–	6·80E-06	4·05E-02	0·044	0·229
ILMN_1702636	3779579	*84617*	*TUBB6*	MESA	minO2	–	2·02E-06	3·29E-03	0·032	0·908
ILMN_1699489	3779579	*84617*	*TUBB6*	MESA	minO2	–	1·47E-04	4·55E-02	0·032	0·908
ILMN_1717877	2448073	*10625*	*IVNS1ABP*	MESA	minO2	+	7·71E-06	9·12E-03	0·018	0·144
ILMN_2397750	2448073	*10625*	*IVNS1ABP*	MESA	minO2	+	2·64E-06	3·60E-03	0·018	0·144
ILMN_2235283	3954238	*5594*	*MAPK1*	MESA	minO2	–	1·43E-06	2·85E-03	0·037	0·830
ILMN_2058251	3236958	*7431*	*VIM*	MESA	avgO2	–	7·10E-05	4·72E-02	0·002	0·823
ILMN_1747775	3478457	*2054*	*STX2*	MESA	avgO2	+	1·39E-05	2·33E-02	0·008	0·568
ILMN_1656920	3554851	*1396*	*CRIP1*	MESA	minO2	–	7·97E-05	3·48E-02	0·037	0·198

**Fig. 2:**
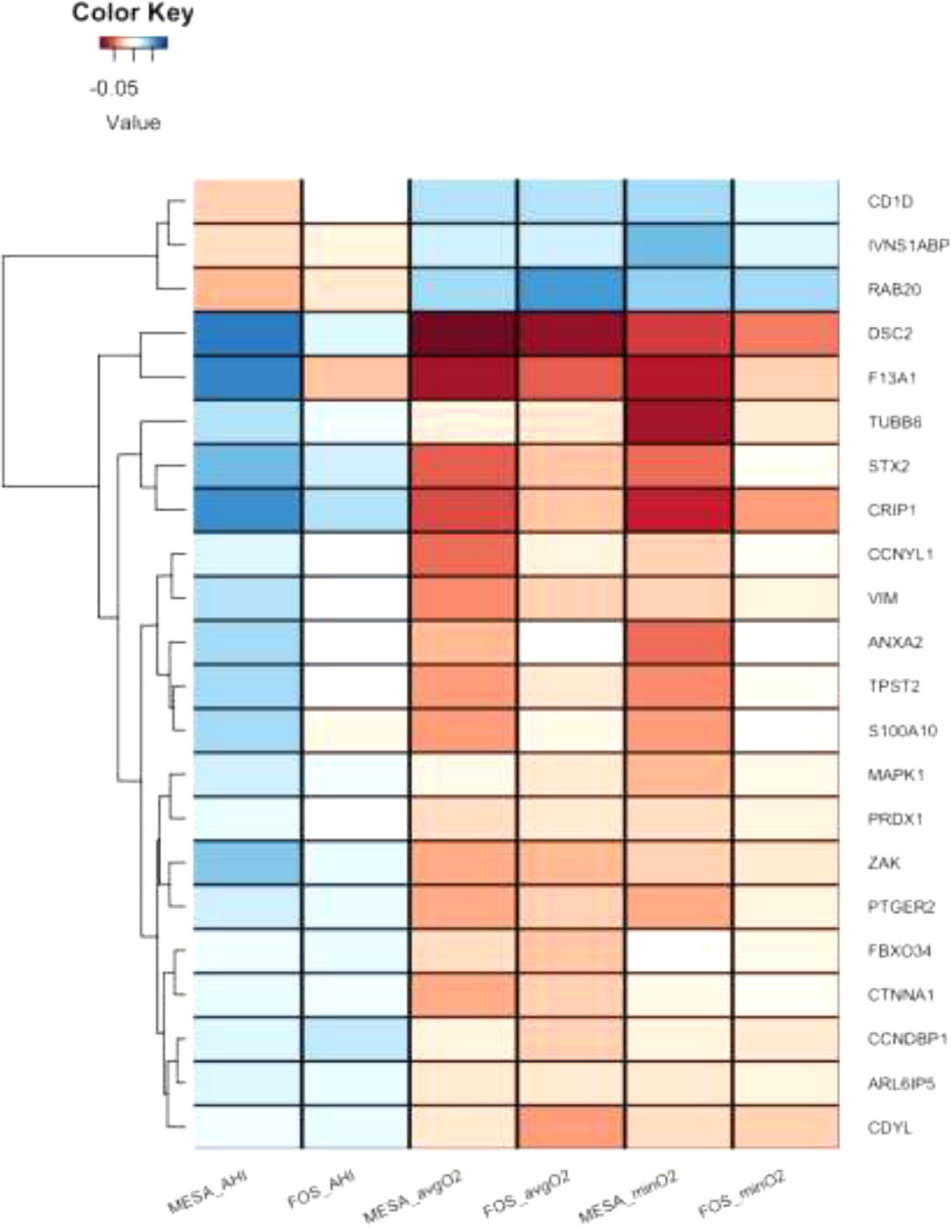
**Clustered heatmap of standardized effect sizes for the SDB-associated genes in each of the SDB traits**. For each of the genes that cross-replicated in MESA and FOS, or were discovered in their meta-analysis, the figure displays the effect sizes per 1 standard deviation of the trait, in the study. In MESA, effect sizes are based on the meta-analysis of the estimates in the three race/ethnic groups. Positive (blue) effect sizes of AHI and negative (orange) effect sizes of avgO2 and minO2 represent higher expression with more severe SDB symptoms. When multiple probes interrogated a single gene, the figure displays only one of them, selected at random, because the results were similar across all transcripts within each gene. (For interpretation of the references to color in this figure legend, the reader is referred to the web version of this article.)

Table 3 lists 21 genes whose expressions was associated with either AHI, avgO2, or minO2 in the meta-analysis of FOS and MESA. Of these genes, 14 (67%) were significant only in the meta-analysis, including *CD1D*, whose expression was lower in association with more severe OSA in the discovery cohorts and significantly increased with CPAP in the HeartBEAT study (*p* = 0·002). Fig. 2 provides a clustered heatmap of the effect sizes (in standard deviations) of the identified transcripts that either cross-replicated between FOS and MESA, or were detected in the meta-analysis for each of the SDB traits.

**Table 3:** Genes identified in the meta-analysis of FOS and MESA. Each row represents a gene transcript that was identified (FDR *q <* 0·05) in the meta-analysis of FOS and MESA trait-specific results. The identifiers corresponding to the transcript within each gene expression platform (MESA probe, FOS probeset, HeartBEAT Entrez Gene ID) are indicated. Meta-analysis *p* values and FDR *q* values are the original and the FDR *q* values, respectively, computed on the meta-analysis results of the relevant trait. HeartBEAT replication *p* values are one-sided *p* values guided by the direction of association in the meta-analysis. Sign is the direction of estimated association, with a positive (negative) sign representing higher (lower) expression with increasing trait value. Complete summary statistics from association analysis are provided in Supplementary files E5 (meta-analysis of FOS and MESA), and E6 (HeartBEAT). Fig. 2 visualizes the effect sizes for these transcripts in MESA and FOS for all investigated traits.

MESA Probe	FOS Probeset	HeartBEAT Entrez Gene ID	Gene	Trait	Sign	Meta-Analysis *p*	Meta-Analysis FDR *q*	HeartBEAT replication *p*
ILMN_1708881	3525498	55647	*RAB20*	avgO2	+	1·93E-08	2·14E-04	0·046
ILMN_2381257	3802980	1824	*DSC2*	avgO2	–	3·26E-07	1·78E-03	0·999
ILMN_2397750	2448073	10625	*IVNS1ABP*	minO2	+	5·51E-07	5·56E-03	0·144
ILMN_1663119	3802980	1824	*DSC2*	avgO2	–	6·13E-07	2·27E-03	0·999
ILMN_2235283	3954238	5594	*MAPK1*	minO2	–	9·52E-07	5·56E-03	0·830
ILMN_1717877	2448073	10625	*IVNS1ABP*	minO2	+	1·54E-06	5·78E-03	0·144
ILMN_1702247	3591327	23582	*CCNDBP1*	AHI	+	1·99E-06	2·43E-02	0·944
ILMN_2046730	2435383	6281	*S100A10*	minO2	–	2·01E-06	5·87E-03	0·949
ILMN_1678075	2892979	9425	*CDYL*	avgO2	–	2·43E-06	5·44E-03	0·229
ILMN_1747775	3478457	2054	*STX2*	avgO2	+	4·34E-06	6·86E-03	0·568
ILMN_1796712	2435383	6281	*S100A10*	avgO2	–	4·86E-06	7·11E-03	0·949
ILMN_2058251	3236958	7431	*VIM*	avgO2	–	5·19E-06	7·25E-03	0·823
ILMN_1768110	2515933	51776	*ZAK*	avgO2	–	5·94E-06	7·53E-03	0·987
ILMN_2230902	2830946	1495	*CTNNA1*	avgO2	–	6·91E-06	7·82E-03	0·847
ILMN_1796712	2435383	6281	*S100A10*	minO2	–	7·35E-06	1·72E-02	0·949
ILMN_2046730	2435383	6281	*S100A10*	avgO2	–	7·96E-06	8·08E-03	0·949
ILMN_1765060	3536786	55030	*FBXO34*	avgO2	–	8·02E-06	8·09E-03	0·416
ILMN_1656920	3554851	1396	*CRIP1*	minO2	–	1·67E-05	3·23E-02	0·198
ILMN_1719433	2362157	912	*CD1D*	avgO2	+	1·85E-05	1·68E-02	0·002
ILMN_1755937	3627248	302	*ANXA2*	minO2	–	2·52E-05	4·21E-02	0·860
ILMN_1782538	3236958	7431	*VIM*	avgO2	–	3·41E-05	2·69E-02	0·823
ILMN_1717163	2940202	2162	*F13A1*	avgO2	–	4·85E-05	3·41E-02	0·846
ILMN_1810069	2525182	151195	*CCNYL1*	avgO2	–	5·38E-05	3·64E-02	0·950
ILMN_2329679	3955915	8459	*TPST2*	avgO2	–	6·91E-05	4·21E-02	0·122
ILMN_1769810	2628682	10550	*ARL6IP5*	avgO2	–	6·95E-05	4·22E-02	0·824
ILMN_1703926	3535780	5732	*PTGER2*	avgO2	–	7·56E-05	4·42E-02	0·516
ILMN_2366391	2410241	5052	*PRDX1*	avgO2	–	7·82E-05	4·50E-02	0·885
ILMN_1717877	2448073	10625	*IVNS1ABP*	avgO2	+	8·42E-05	4·67E-02	0·144

### 3.3 Sensitivity analysis adjusting for BMI

In a sensitivity analysis reported in Supplementary Tables E2 and E3, most of the associations become slightly weaker after adjustment for BMI; however, BMI directly influences SDB by altering airway collapsibility, and it is therefore not clear that adjustment for BMI results in a less rather than a more confounded effect estimate. The response to CPAP therapy of *CD1D* and *RAB20* suggests that the expression changes identified for these genes are due to OSA and not obesity. Notably, the BMI of HeartBEAT individuals slightly increased with CPAP treatment, from an average of 31.4 before to 31.9 after CPAP treatment.

### 3.4 Pathway-based identification of processes altered in SDB

We applied Gene Set Enrichment Analysis (GSEA) to the entire available transcriptome in each cohort. We surveyed 1379 canonical pathways and applied a strict FDR significance threshold (FDR *q <* 0·05). Supplementary file E1 provides complete results and Table E1 in the Supplementary Materials provides the number of significant pathways (FDR *q* < 0·05) in each of the analyses. Supplementary fig. E1 visualizes all pathways with FDR *q <* 0·05 in one analysis and *p* < 0·05 in at least one other analysis.

**Fig. 3:**
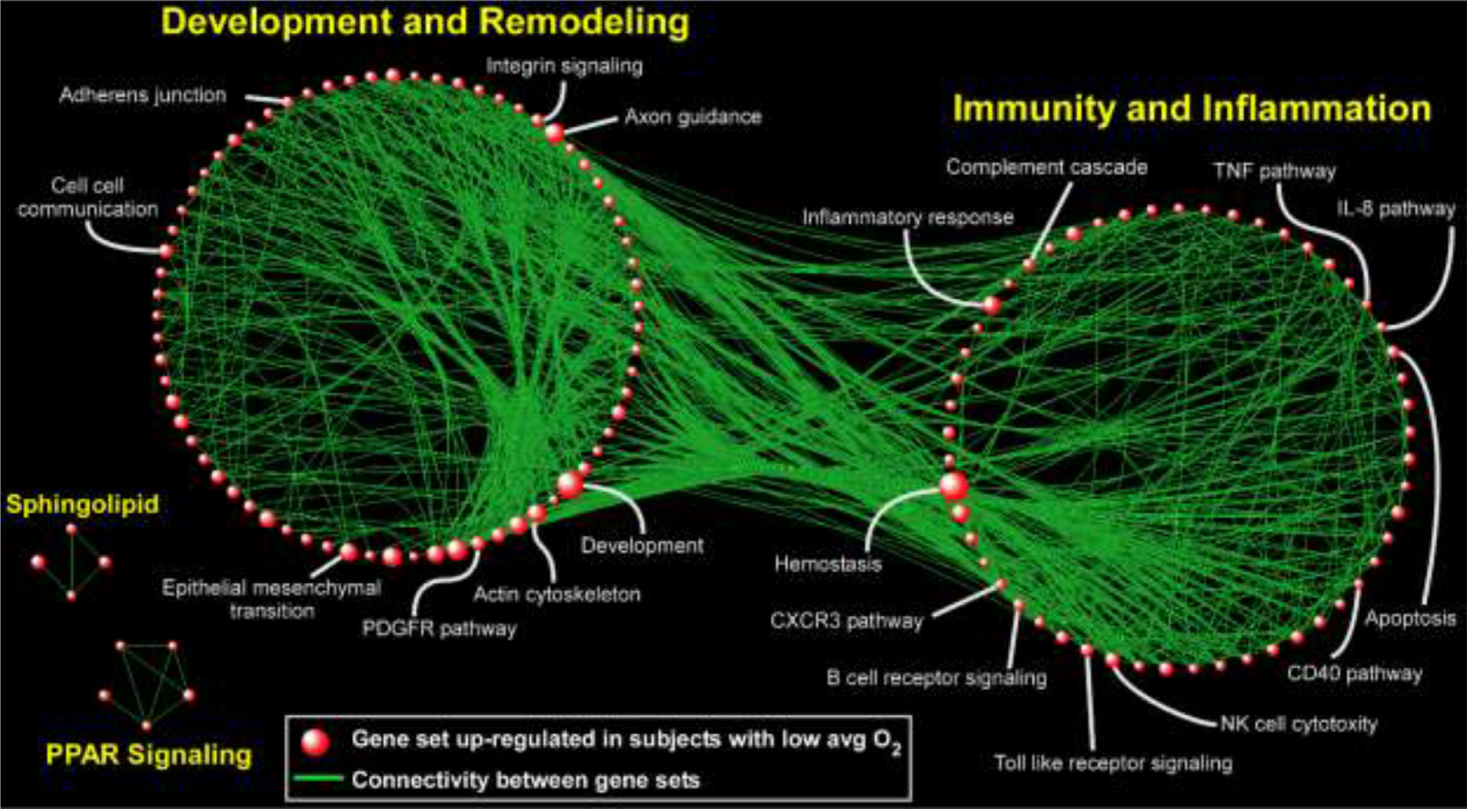
**Network-based depiction of up-regulated gene sets in the analysis of gene expression association with lower avgO2 (i.e., more severe SDB) in MESA**. Gene sets with FDR *q <* 0•05 are shown. The sizes of the symbols correspond to the number of genes in the set. Connectivity is based on overlapping genes among gene sets. Note that we did not identify any gene sets that were down-regulated (FDR *q* < 0•05) in association with more severe SDB.

**Fig. 4:**
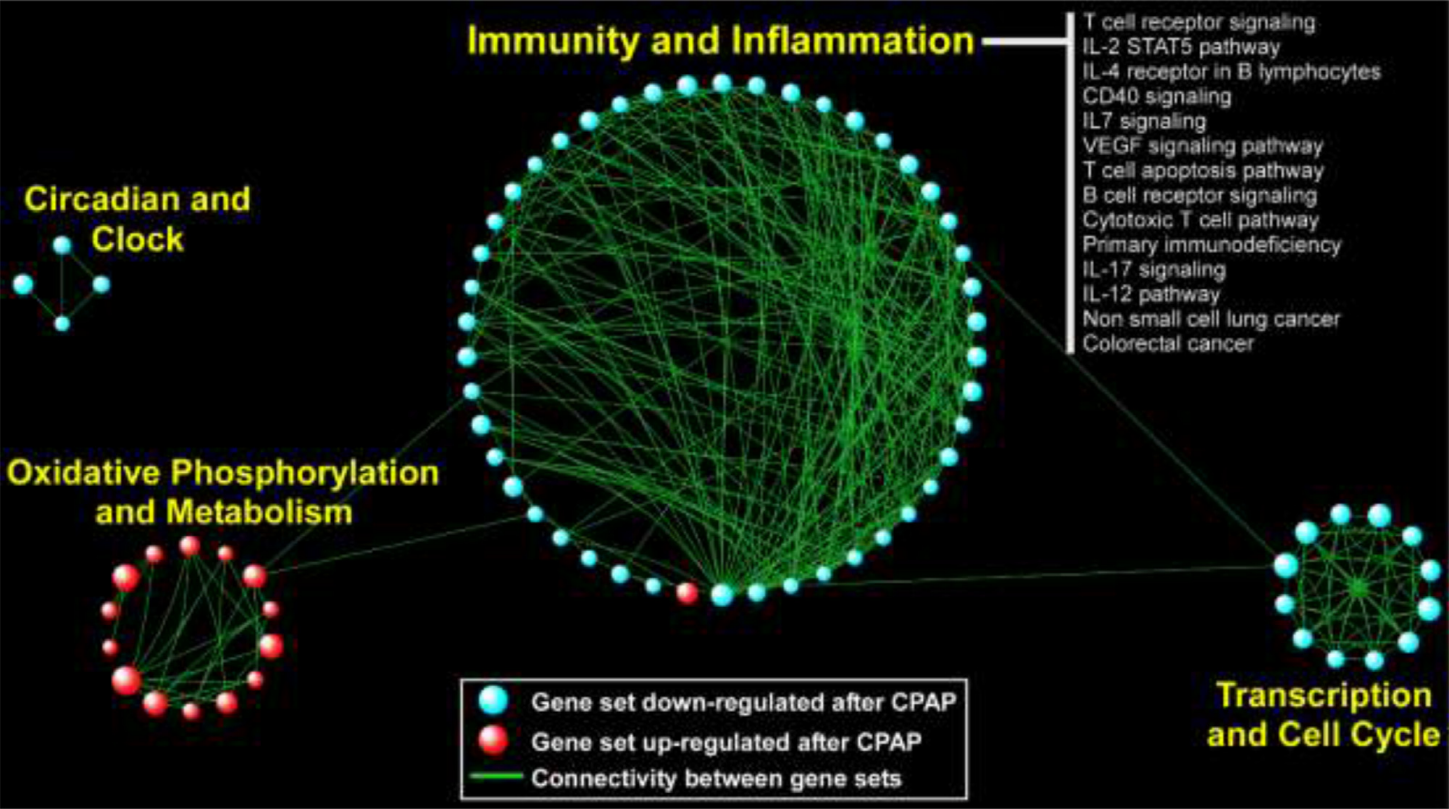
**Network visualization of up- and down-regulated gene sets detected in the HeartBEAT study**. Gene sets with FDR *q <* 0•05 are shown. The sizes of the symbols correspond to the number of genes in the set. Connectivity is based on overlapping genes between gene sets.

In the FOS study, in which gene expression was assayed several years after the sleep study, heme metabolism was strongly and significantly up-regulated with respect to SDB severity; this was also observed in MESA, although it did not pass multiple testing correction (AHI *p* = 0·02; avgO2 *p* = 0·002). Other pathways had less consistent patterns across analyses. In MESA, the strongest signal was found for avgO2: 150 gene sets were significantly up-regulated (FDR *q*<0·05) with low average oxyhemoglobin saturation (*i.e*., with worse SDB). A visual summary of this result is depicted in Fig. 3, where network analysis was used to link related gene sets into larger modules comprised of functionally similar processes, in particular those related to “Immunity and Inflammation” and “Development and Remodeling”. In the HeartBEAT cohort, we found that pathways involved in immunity and inflammation, transcription and cell cycle, and circadian rhythms were down-regulated following CPAP therapy, whereas gene sets related to oxidative phosphorylation and metabolism were up-regulated (Fig. 4). Taken together, our pathway analyses across multiple cohorts suggested that immuno-inflammatory processes are activated in individuals with low avgO2 and, importantly, effective treatment of OSA with CPAP reverses this pro-inflammatory signal.

## 4 Discussion

We performed a comprehensive study of the association of SDB traits with genome-wide gene expression in peripheral blood. We combined two large population-based studies, FOS and MESA, to study the associations of three continuous measures of SDB in the general population, and we also compared gene expressions before and after CPAP treatment in a subset of individuals from the HeartBEAT clinical trial who had moderate to severe OSA and were adherent to treatment. We identified 22 genes whose expressions were associated with SDB traits in either cross-replication or meta-analysis of FOS and MESA. Most of these differentially expressed genes were associated with measures of hypoxemia: avgO2 and minO2. While these measures of hypoxemia may be influenced by conditions other than SDB, two of these genes, *CD1D* and *RAB20*, showed evidence of change with OSA treatment in the expected directions; both were expressed at lower levels in individuals with more severe SDB in FOS and MESA, and their expression increased significantly in HeartBEAT patients following treatment with CPAP, suggesting that OSA was the cause of their differential expression.

The *RAB20* gene encodes the Ras-related protein Rab20, a member of the Rab family of small GTPAses. Expression of *RAB20* is regulated by Hypoxia-Inducible transcription factor (HIF-1*α*; [33]), a protein whose expression responds to changes in tissue oxygen tension, and enhances oxygen availability and reduces oxygen demand in a hypoxic microenvironment [34]. In our analysis, more severe SDB (higher AHI, lower minO2, and avgO2) was associated with lower expression of *RAB20*. Impaired *RAB20* expression in human skeletal muscle is associated with impaired skeletal muscle glucose uptake and reduced total body insulin sensitivity [34]. Reduced insulin sensitivity is a common pathophysiological finding in SDB [35] and can be induced by as little as 8 h of exposure to intermittent hypoxia [36]. Recent animal data also indicate that *RAB2*0 is a negative regulator of neurite outgrowth, and thus may influence neuronal network development, potentially influencing brain development [37]. The lower *RAB20* expression in association with SDB severity is somewhat surprising, as hypoxemia is reportedly associated with HIF-1-mediated up-regulation of *RAB20* expression [33]. There was only a very weak evidence for association of *HIF-1α* expression (see Supplementary file E5) with SDB traits in our analysis, however, possibly reflecting the complexity of pathways that regulate HIF-1, including both hypoxia and redox-sensitive pathways, as well as the predominantly post-transcriptional mechanisms regulating HIF-1 signaling [38]. That CPAP treatment was associated with increased *RAB20* expression in HeartBEAT subjects implies that OSA was the cause of reduced *RAB20* expression in the cross-sectional analysis, and suggests a mechanism whereby CPAP treatment may improve glucose metabolism in OSA patients.

The *CDID* gene encodes the CD1D antigen, an MHC class I-like molecule expressed on both myeloid and lymphoid cells that binds glycolipids and presents them to T cell receptors on natural killer T cells [39,40]. It is possible that reduction in CD1D antigen, potentially associated with the observed reduction in its expression, may contribute to impaired immune function that has been observed in individuals with OSA [41,42]. *CD1D* expression was reported to be regulated by retinoic acid in mononuclear cells [43]. In addition, the retinoic acid pathway was significantly down-regulated in lower avgO2 in MESA, and up-regulated in response to CPAP treatment in HeartBEAT (Supplementary File E1), suggesting a role for retinoic acid in mediating reduced CD1D expression in SDB. It has also been reported that natural killer (NK) T cells have impaired maturation and cytotoxicity in untreated OSA patients, and that this immunosuppressive phenotype was correlated with severity of hypoxemia [44].

Other genes identified by the FOS and MESA analysis that did not demonstrate a response to CPAP treatment in HeartBEAT subjects include *MAPK1*, involved in the HIF-1*α* response to hypoxia [45], and *CTNNA1*, that encodes a protein shown to respond to chronic intermittent hypoxia in a mouse study of transcription in cardiac rhythm genetic networks [45]. Two related genes were up-regulated under lower oxygen saturation: annexin A2 (*ANXA2*) and S100 Calcium Binding Protein A10 (*S100A10*). These genes code proteins that interact with each other [46] and both were shown to contribute to pulmonary microvascular integrity by preventing vascular leak during alveolar hypoxia in mice [47]. Since the expression of these genes did not respond to CPAP therapy, they may indicate persistent alterations in transcriptional response as a result of chronic OSA.

Hypoxemia is known to influence the expression of a wide array of genes and prior studies have demonstrated many more genes are up-regulated than down-regulated in response to experimental sustained or intermittent hypoxemia [48]. Consistent with this finding, we observed many more pathways up-regulated than down-regulated in association with increased severity of SDB traits such as low avgO2. Gene set enrichment analysis demonstrated up-regulation of heme metabolic pathways across FOS and MESA cohorts with more severe SDB. Previous experiments showed that subjecting cells to hypoxia up-regulates the expression of several proteins involved in iron/heme metabolism, in order to increase erythropoiesis and hematopoietic iron supply [49,50]. Our previous genetic association analysis identified variants eQTL for ferrochelatase (*FECH*), the terminal enzyme in heme biosynthesis, associated with SDB traits [51], suggesting a possible bidirectional association between SDB and heme metabolism pathways. Turnbull et al. [15] in a CPAP withdrawal study found that the heme metabolic pathway was upregulated after two weeks of CPAP withdrawal into both supplemental air (sham) and supplemental oxygen. This suggest that the SDB effect on heme metabolic pathway may occur via non-hypoxia mechanisms. More work is needed to elucidate the mechanisms behind this association.

A key finding of our study is that in a large, ethnically diverse cohort (MESA), SDB is associated with up-regulation of pathways involved in immune function and inflammation (Fig. 3). Further, we found that this immuno-inflammatory signal was down-regulated in OSA patients following CPAP treatment (Fig. 4), providing transcriptome-wide evidence that is consistent with a reversal of known pro-inflammatory markers of OSA with effective therapy [52]. Our results agree with the results from a recent transcriptomics analysis following CPAP withdrawal [15]. Another large biological module emerging from pathway analysis of the MESA cohort was up-regulation of gene sets involved in development and remodeling. This finding is supportive of the growing evidence based on animal models and human data that OSA and nocturnal hypoxemia are associated with aberrant cardiovascular remodeling [53,54]. In the HeartBEAT cohort, we also observed down-regulation of pathways involved in cancer and cell cycle following resolution of OSA and nocturnal hypoxemia with CPAP therapy. Although many cell types, including lymphocytes, may undergo cell cycle arrest in response to hypoxia [54,55], our findings are consistent with recent work supporting down-regulation of neoplastic programs after CPAP [13] and the potentially increased risk of cancer associated with OSA and nocturnal hypoxemia [56,57].

In this work, we utilized data from three different studies to identify genes and gene sets that show expression levels that vary with SDB traits, then explored which expression signals change with OSA treatment. These studies differed in designs and populations, resulting in several important limitations. Different ethnic composition between the cohorts and the time lag between PSG and blood draw in MESA (on average, blood was draw 299 days before the sleep study) and FOS (sleep study preceded blood draw by 2–10 years) reduce the statistical power to detect associations. Reliance on a single sleep study, rather than averaging the result of multiple nights, likely reduces the accuracy of sleep measures as a reflection the average chronic exposure to SDB over time. While all indices may be sensitive to nigh-to-night variation, we have found them to be heritable [19,51], suggesting that despite measurement error and physiological variability, there is significant between individual biological variation captured by the single night recording. This is consistent with the extensive epidemiologic literature demonstrating an association of measures from a single night PSG with SDB symptoms and both prevalent and incident cardiovascular and metabolic disease. The use of different blood cell types (monocytes in MESA, whole blood in FOS, and mononuclear cells in HeartBEAT) may also have reduced the ability to replicate findings across cohorts. Each of these limitations would bias towards a null result; therefore, while it is likely that we failed to identify some true gene expression effects of SDB, these limitations should not have introduced spurious positive findings. Another limitation of our study is that measures of oxygen saturation (avgO2 and minO2) are not specific to SDB and can be affected by pulmonary and cardiac function; however, the reversal of gene expression changes by CPAP treatment indicates that SDB, rather than other disorders, was the likely cause of the gene expression changes in *RAB40* and *CD1D* observed in MESA and FOS. Notably, despite differences in the temporal associations between the sleep studies and gene expression assays, and racial/ethnic composition of the samples, we observed consistent associations for 22 genes in FOS and MESA. The comparability of findings despite variations in the timing between the sleep phenotyping and blood draws may reflect that relative stability of SDB traits over time [58]. In the Supplementary Material, we further report look-ups of previously-reported genes associated with OSA and with CPAP treatment.

In summary, we report the largest gene expression analysis of SDB traits to date. While precise measures of SDB are challenging to obtain in large-scale cohorts, we leveraged the availability of population-based studies that performed in-home polysomnography and combined our transcriptional analysis of these cohorts with a clinical trial for better control of confounding and confirmation of expression changes with treatment. Using a gene-focused discovery and validation methodology, we identified *CD1D* and *RAB20* as being key down-regulated genes in SDB whose expression responded to CPAP treatment. A complementary pathway-based approach revealed that SDB severity is associated with alteration of distinct peripheral blood programs including up-regulation of immuno-inflammatory signals that were reversed with effective CPAP therapy. Collectively, our study provides a framework to investigate the transcriptional consequences of SDB traits in multi-cohort datasets and identify putative candidate genes and processes driving this response.

## Declarations of Competing Interest

Dr. Rotter reports grants from NIH, during the conduct of this study. Dr. Redline reports grants from NIH,  during the conduct of the study. Dr. Patel reports grants from Bayer Pharmaceuticals, Philips Respironics, and Respicardia outside the submitted work. Dr. Mehra reports receiving National Institutes of Health funding support from the National Heart, Lung, and Blood Institute [U01HL125177, UG3HL140144] and the American Heart Association. Her institution has received positive airway pressure devices and equipment from Philips Respironics, ResMed, GE Healthcare, and Natus for research. R. M serves as a consultant for Respicardia, Enhale and Merck; received funds for service on the American Board of Medicine Sleep Medicine Exam test writing committee and received royalties from UpToDate. Dr. Gottlieb reports grants from NIH/NHLBI,  during the conduct of the study. Dr. Quan reports personal fees from Jazz Pharmaceuticals, personal fees from Best Doctors, other from Sleep Research Society, personal fees from American Academy of Sleep Medicine,  outside the submitted work.

## Appendix Supplementary materials

Image, application 1

Image, application 2

Image, application 3

Image, application 4

Image, application 5

Image, application 6

Image, application 7
