# Supplementary material for: Low oxygen saturation during sleep reduces CD1D and RAB20 expressions that are reversed by CPAP therapy
Source: eBioMedicine. 2020 Jun 5;56:102803. doi: 10.1016/j.ebiom.2020.102803 (PMC7276515; doi:10.1016/j.ebiom.2020.102803)
Supplement: Supplementary file 7 [file mmc7.docx]

**Low oxygen saturation during sleep reduces CD1D and RAB20 expressions that are reversed by CPAP therapy: Online Supplement**

Tamar Sofer, Ruitong Li, Roby Joehanes, Honghuang Lin, Adam C. Gower, Heming Wang, Nuzulul Kurniansyah, Brian E. Cade, Jiwon Lee, Stephanie Williams, Reena Mehra, Sanjay R. Patel, Stuart F. Quan, Yongmei Liu, Jerome I. Rotter, Stephen S. Rich, Avrum Spira, Daniel Levy, Sina A. Gharib, Susan Redline, Daniel J. Gottlieb

[Supplementary Methods and Results 2](#_Toc37273198)

[Gene expression data collection and processing in MESA 2](#_Toc37273199)

[Gene expression data collection and processing in FOS 3](#_Toc37273200)

[Report of look-up of previously-reported associations in the current analyses 3](#_Toc37273201)

[Supplementary Tables 4](#_Toc37273202)

[Supplementary Figures 9](#_Toc37273203)

[Description of additional supplementary files 10](#_Toc37273204)

[Data and materials availability 11](#_Toc37273205)

# Supplementary Methods and Results

## Gene expression data collection and processing in MESA

Gene expression profiling in MESA was described in Liu et al (1). Briefly, peripheral blood samples of 1,264 randomly selected MESA participants from four MESA field centers (Baltimore, MD; Forsyth County, NC; New York, NY; and St Paul, MN) were obtained during the fifth examination. Blood was drawn in the morning following 8 hours fast. Blood collection and monocyte sample purification were performed sequentially using sodium heparin-containing Vacutainer CPT_TM_ cell separation tubes (Becton Dickinson, Rutherford, NJ, USA) and anti-CD14-coated magnetic beads, using an AutoMACs automated magnetic separation unit (Miltenyi Biotec, Bergisch Gladbach, Germany). mononuclear cells were isolated with consistent purity of > 90%. Extracted RNA that passed Quality Control (QC) testing was reversely transcribed, amplified and subjected to global expression microarrays. Global expression quantification was measured by Illumina HumanHT-12 v4 Expression BeadChip and Illumina Bead Array Reader. Samples were assigned to chips via stratified random sampling to prevent potential batch, chip, and position biases. Gene expression data underwent pre-processing, QC, and normalization, in steps including local background correction, elimination of probes not satisfying QC criteria, and normal-exponential convolution model. This was done using a series of Bioconductor packages (beadarray, limma etc) and Illumina software GenomeStudio. These steps resulted in gene expression data for 14,619 transcripts for further statistical analysis.

## Gene expression data collection and processing in FOS

Gene expression profiling and consecutive processing for FOS cohort have been described in detail by Joehanes et al and Lin et al (2, 3). Briefly, collected peripheral whole blood samples from each participant were used for RNA extraction. Total RNA was then amplified, labeled, and reversely transcribed into cDNA. The cDNA hybridization was conducted on a Human Exon 1.0 ST array. Pre-processing steps included log2 transformation, quantile normalization, and summarization with Robust Multi-array Average method. QC steps excluded exons with low signal to noise ratio and transcript clusters with missing matches in RefSeq transcript records. This resulted in 17,873 distinct transcripts available for analysis. Subsequent statistical analysis was adjusted for multiple batch effects and technical variables and estimated white cell counts as described in (2).

## Report of look-up of previously-reported associations in the current analyses

Other studies performed gene expression association analysis in adults using peripheral blood. In data file S2 we provide tables in which we report the association analysis results in MESA, FOS, and HeartBEAT, for the genes reported by Chen et al. (2017) (4) and by Perry et al. (2013) (5) as either differentially expressed between OSA patients and controls, or additionally responding to CPAP treatment. Chen et al. reported genes with expression responding to a 1-year CPAP treatment. Of these, *GAS6*, *LGALS3*, and *NACC2* had some evidence of association in MESA and FOS (at least one trait with *p* < 0.01), and *LGALS3* also has some evidence of change by CPAP treatment in HeartBEAT (*p* = 0.04). However, the directions of association did not always match between Chen et al. and our study. Perry et al. reported gene expression changes between individuals with OSA and control individuals, and response to 6-months CPAP treatment among severe OSA individuals. Genes that had *p* < 0.01 in at least one of the traits in MESA and/or FOS are: *CAT*, *CSTB*, *HIF1A*, and *PRPF40A*. *CAT* had *p* = 0.03 in HeartBEAT, but it became more highly expressed after CPAP treatment, rather than less expressed as seen in Perry et al. and opposite the change expected based on increased expression with more severe SDB in MESA and FOS. Inconsistent results between studies may be due to both noise, and low statistical power.

# Supplementary Tables

Table E1. **Number of gene sets with significant GSEA results (FDR *q* < 0.25) in each of the cohort and trait analyses.**

| **Cohort** | **SDB trait association** | **Number of significant gene sets** |
| --- | --- | --- |
| MESA | Up-regulated with higher AHI | 10 |
|  | Up-regulated with lower AHI | 2 |
|  | Up-regulated with higher avgO2 | 2 |
|  | Up-regulated with lower avgO2 | 150 |
|  | Up-regulated with higher minO2 | 0 |
|  | Up-regulated with lower minO2 | 0 |
| FOS | Up-regulated with higher AHI | 1 |
|  | Up-regulated with lower AHI | 2 |
|  | Up-regulated with higher avgO2 | 0 |
|  | Up-regulated with lower avgO2 | 1 |
|  | Up-regulated with higher minO2 | 0 |
|  | Up-regulated with lower minO2 | 1 |
| HeartBEAT | Up-regulated after CPAP | 17 |
|  | Down-regulated after CPAP | 62 |

Table E2. **P-values from association analyses in MESA for transcripts identified in cross-replication (reported in Table 2) analysis in four nested models**: model 1 (primary) adjusts for age, sex, visit site, cell enrichment scores and gene expression chip; model 2 further adjusts for BMI; model 3 additionally adjust for smoking status, and model 4 additionally adjusted for alcohol intake status.

| **MESA Probe** | **Trait** | **Gene** | **P-value** | | | | |
| --- | --- | --- | --- | --- | --- | --- | --- |
|  |  |  | **model 1** | **model 2** | **model 3** | **model 4** |  |
| ILMN_1708881 | aveO2 | RAB20 | 7.41E-02 | 6.61E-01 | 9.57E-01 | 9.05E-01 |  |
| ILMN_1678075 | aveO2 | CDYL | 8.73E-02 | 1.09E-01 | 5.04E-02 | 4.51E-02 |  |
| ILMN_1702636 | minO2 | TUBB6 | 2.02E-06 | 3.69E-04 | 3.61E-04 | 4.52E-04 |  |
| ILMN_1699489 | minO2 | TUBB6 | 1.47E-04 | 4.19E-03 | 4.65E-03 | 4.56E-03 |  |
| ILMN_1717877 | minO2 | IVNS1ABP | 7.71E-06 | 1.95E-02 | 1.32E-02 | 1.23E-02 |  |
| ILMN_2397750 | minO2 | IVNS1ABP | 2.64E-06 | 4.08E-03 | 3.04E-03 | 3.27E-03 |  |
| ILMN_2235283 | minO2 | MAPK1 | 1.43E-06 | 2.10E-04 | 7.68E-04 | 5.56E-04 |  |
| ILMN_2058251 | aveO2 | VIM | 7.10E-05 | 4.07E-04 | 8.68E-04 | 9.02E-04 |  |
| ILMN_1747775 | aveO2 | STX2 | 1.39E-05 | 2.24E-03 | 2.20E-03 | 4.90E-03 |  |
| ILMN_1656920 | minO2 | CRIP1 | 7.97E-05 | 6.55E-03 | 6.48E-03 | 6.04E-03 |  |

Table E3. **P-values from association analyses in MESA for transcripts identified in meta-analysis (reported in Table 3) analysis in four nested models**: model 1 (primary) adjusts for age, sex, visit site, cell enrichment scores and gene expression chip; model 2 further adjusts for BMI; model 3 additionally adjust for smoking status, and model 4 additionally adjusted for alcohol intake status.

| **MESA Probe** | **Trait** | **Gene** | **P-value** | | | | |
| --- | --- | --- | --- | --- | --- | --- | --- |
|  |  |  | **model 1** | **model 2** | **model 3** | **model 4** |  |
| ILMN_1719433 | aveO2 | CD1D | 1.67E-03 | 1.45E-01 | 2.00E-01 | 1.71E-01 |  |
| ILMN_1708881 | aveO2 | RAB20 | 7.41E-02 | 6.61E-01 | 9.57E-01 | 9.05E-01 |  |
| ILMN_2329679 | aveO2 | TPST2 | 2.00E-04 | 4.81E-02 | 6.59E-02 | 4.92E-02 |  |
| ILMN_1717877 | aveO2 | IVNS1ABP | 7.16E-04 | 5.38E-02 | 1.53E-01 | 1.24E-01 |  |
| ILMN_1717877 | minO2 | IVNS1ABP | 7.71E-06 | 1.95E-02 | 1.32E-02 | 1.23E-02 |  |
| ILMN_2397750 | minO2 | IVNS1ABP | 2.64E-06 | 4.08E-03 | 3.04E-03 | 3.27E-03 |  |
| ILMN_1656920 | minO2 | CRIP1 | 7.97E-05 | 6.55E-03 | 6.48E-03 | 6.04E-03 |  |
| ILMN_1678075 | aveO2 | CDYL | 8.73E-02 | 1.09E-01 | 5.04E-02 | 4.51E-02 |  |
| ILMN_1765060 | aveO2 | FBXO34 | 5.82E-03 | 2.36E-03 | 4.03E-04 | 5.51E-04 |  |
| ILMN_1703926 | aveO2 | PTGER2 | 3.12E-04 | 2.06E-03 | 6.19E-04 | 6.07E-04 |  |
| ILMN_1747775 | aveO2 | STX2 | 1.39E-05 | 2.24E-03 | 2.20E-03 | 4.90E-03 |  |
| ILMN_1782538 | aveO2 | VIM | 1.72E-03 | 3.30E-03 | 3.77E-03 | 7.80E-03 |  |
| ILMN_2058251 | aveO2 | VIM | 7.10E-05 | 4.07E-04 | 8.68E-04 | 9.02E-04 |  |
| ILMN_1769810 | aveO2 | ARL6IP5 | 9.08E-04 | 6.11E-02 | 6.08E-02 | 4.95E-02 |  |
| ILMN_2235283 | minO2 | MAPK1 | 1.43E-06 | 2.10E-04 | 7.68E-04 | 5.56E-04 |  |
| ILMN_1717163 | aveO2 | F13A1 | 9.88E-04 | 1.21E-02 | 6.98E-02 | 8.78E-02 |  |
| ILMN_2230902 | aveO2 | CTNNA1 | 1.09E-04 | 1.06E-03 | 7.98E-03 | 7.91E-03 |  |
| ILMN_1755937 | minO2 | ANXA2 | 6.32E-07 | 1.43E-03 | 4.34E-03 | 4.14E-03 |  |
| ILMN_2366391 | aveO2 | PRDX1 | 3.87E-04 | 2.08E-02 | 4.04E-02 | 3.84E-02 |  |
| ILMN_1702247 | AHI | CCNDBP1 | 1.45E-05 | 9.29E-04 | 1.00E-03 | 9.39E-04 |  |
| ILMN_1796712 | aveO2 | S100A10 | 1.66E-06 | 2.93E-04 | 4.38E-05 | 5.27E-05 |  |
| ILMN_1796712 | minO2 | S100A10 | 2.44E-06 | 2.32E-03 | 1.53E-03 | 1.53E-03 |  |
| ILMN_2046730 | aveO2 | S100A10 | 4.21E-06 | 3.77E-04 | 1.54E-04 | 1.64E-04 |  |
| ILMN_2046730 | minO2 | S100A10 | 7.90E-07 | 5.34E-04 | 5.63E-04 | 4.96E-04 |  |
| ILMN_1810069 | aveO2 | CCNYL1 | 3.31E-06 | 2.87E-05 | 4.08E-05 | 3.72E-05 |  |
| ILMN_1768110 | aveO2 | ZAK | 2.55E-03 | 1.63E-02 | 1.26E-02 | 1.64E-02 |  |
| ILMN_1663119 | aveO2 | DSC2 | 4.10E-04 | 6.32E-03 | 4.13E-02 | 4.15E-02 |  |
| ILMN_2381257 | aveO2 | DSC2 | 1.79E-04 | 3.47E-03 | 2.45E-02 | 2.73E-02 |  |

# Supplementary Figures

Figure E1. **Overlap between gene sets with significant GSEA results** (FDR *q <* 0.05 in at least one analysis and *p* < 0.05 in at least one other analysis). Cells are shaded by Normalized Enrichment Score (NES), such that blue, white and red indicate NES values of -3, 0, and 2, respectively.


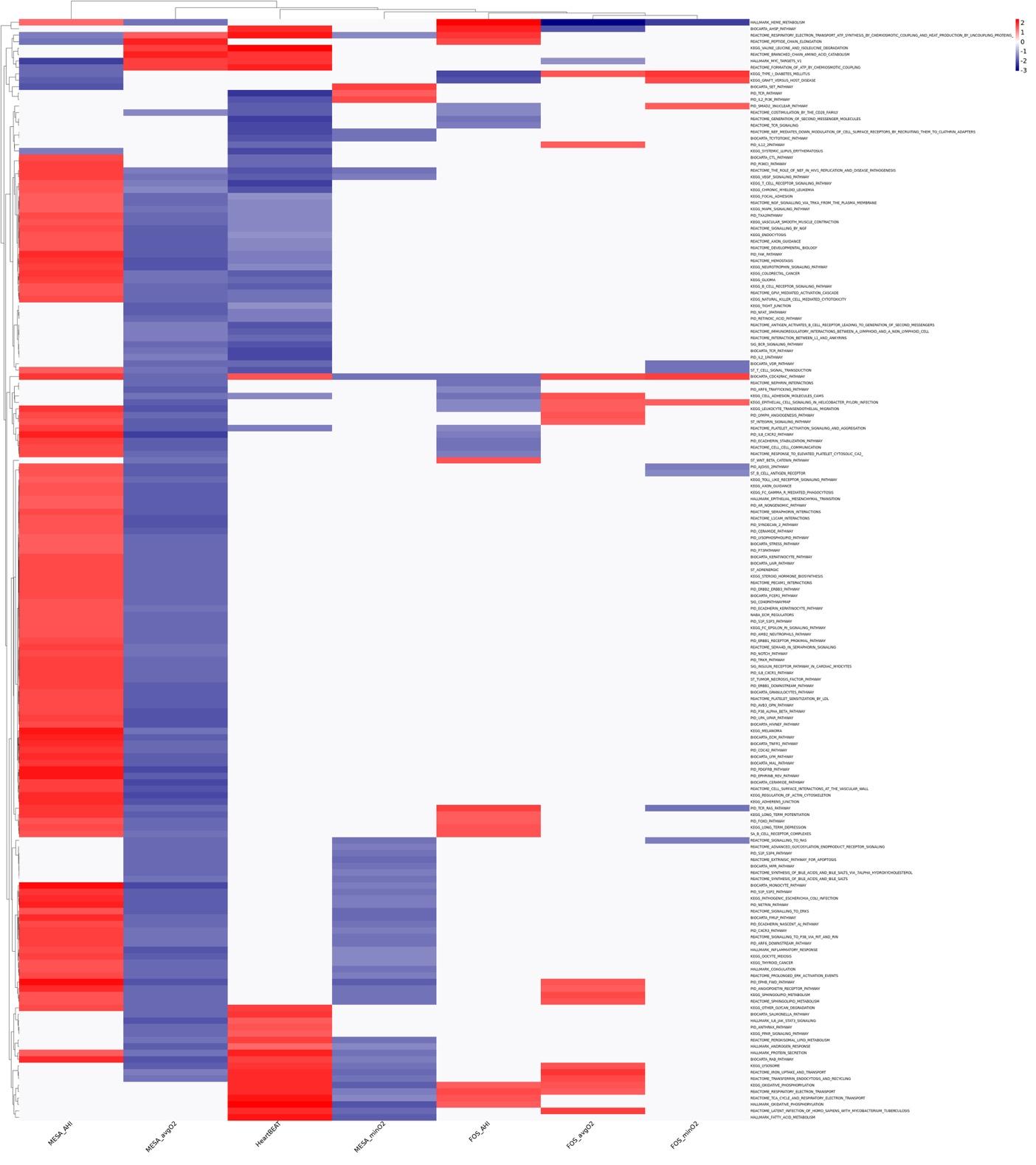


# Description of additional supplementary files

**File E1.** Complete results from Gene Set Enrichment Analyses. For each of the analyses reported in the main manuscript (avgO2, minO2, and AHI in MESA and in FOS, and HeartBEAT change in expression following CPAP treatment), we provide a table with Gene Set Enrichment Analysis (GSEA) results for positive (pos) and negative (neg) enrichment. Each table contains pathway name, number of genes in the pathway (Gene_num), positive and negative enrichment scores, *p* value, and FDR *q* values.

**File E2.** Gene expression associations of previously reported genes. For genes reported in Chen et al. (2017) and in Perry et al. (2013), we report summary statistics from gene expression association testing in FOS, MESA, and HeartBEAT (HB). For MESA and FOS, summary statistics are provided as beta (effect sizes estimate) and *p* value (p) for each of the analyzed traits. For HeartBEAT, effect sizes are given as fold-changes in gene expression following 3 months of CPAP treatment, and *p* value is the usual (two-sided) *p* value based on the *t* test. Genes that had corresponding effects with *p* < 0.1 are color coded by their *p* value.

**File E3.** Complete results from transcriptome-wide association analyses with SDB traits in MESA. Each row provides Illumina probe ID, gene symbol, and estimated effect sizes (Beta), standard errors (SE), *p*-values (pval) and *q*-values (FDRq) for each of the trait-specific analysis.

**File E4.** Complete results from transcriptome-wide association analyses with SDB traits in FOS. Each row provides Affymetrix probeset ID, gene symbol, and estimated effect sizes (Beta), standard errors (SE), *p*-values (pval) and *q*-values (FDRq) for each of the trait-specific analysis.

**File E5.** Complete results from the meta-analyses of transcriptome-wide association analyses in MESA and FOS. Each row provides Illumina probe ID (MESA), Affymetrix probeset ID (FHS), gene symbol, and estimated effect sizes (Beta), standard errors (SE), *p*-values (pval) and *q*-values (FDRq) for each of the trait-specific analysis.

**File E6.** Complete results from transcriptome-wide association analysis with CPAP treatment in HeartBEAT. For each gene, we provide Entreze ID, gene symbol, Beta, which here are the difference in log2-transformed expression values post- and pre-CPAP treatment, pval is the t-test *p*-value, and FDRq is the FDR *q*-value.

# Data and materials availability

All summary statistics from analysis reported in this manuscript are provided in the Supplementary Material. Raw gene expression data for FOS are a part of SABRe CVD study on dbGaP, with dbGaP study accession: phs000363.v17.p11. Data could could be obtained by requesting authorized access to the Framingham Cohort Study phs000007 on dbGaP. Gene expression data for MESA could be obtained using application to the MESA https://www.mesa-nhlbi.org. Raw and processed gene expression data for HeartBEAT have been deposited in the Gene Expression Omnibus (GEO), Series GSE133601.

**References**

1. Liu Y, Ding J, Reynolds LM, Lohman K, Register TC, De La Fuente A, et al. Methylomics of gene expression in human monocytes. Hum Mol Genet. 2013;22(24):5065-74.

2. Joehanes R, Ying S, Huan T, Johnson AD, Raghavachari N, Wang R, et al. Gene expression signatures of coronary heart disease. Arterioscler Thromb Vasc Biol. 2013;33(6):1418-26.

3. Lin H, Yin X, Lunetta KL, Dupuis J, McManus DD, Lubitz SA, et al. Whole blood gene expression and atrial fibrillation: the Framingham Heart Study. PLoS One. 2014;9(5):e96794.

4. Chen YC, Chen KD, Su MC, Chin CH, Chen CJ, Liou CW, et al. Genome-wide gene expression array identifies novel genes related to disease severity and excessive daytime sleepiness in patients with obstructive sleep apnea. PLoS One. 2017;12(5):e0176575.

5. Perry JC, Guindalini C, Bittencourt L, Garbuio S, Mazzotti DR, Tufik S. Whole blood hypoxia-related gene expression reveals novel pathways to obstructive sleep apnea in humans. Respir Physiol Neurobiol. 2013;189(3):649-54.
